# Characterization and typology of goat production systems in the Southern Highlands of Peru

**DOI:** 10.14202/vetworld.2025.220-227

**Published:** 2025-01-30

**Authors:** Emmanuel Alexander Sessarego, Fritz Carlos Trillo, David José Godoy, Walter Palomino-Guerrera, Juancarlos Alejandro Cruz

**Affiliations:** Dirección de Supervisión y Monitoreo en las Estaciones Experimentales Agrarias, Instituto Nacional de Innovación Agraria, Av. La Molina # 1981, Lima, Perú

**Keywords:** Chincha Highlands, extensive farming, goat production systems, multivariate analysis, Peru, typology

## Abstract

**Background and Aim:**

Characterizing local animal production systems is crucial for sustainable livestock development. This study aimed to characterize the diversity of goat production systems in the Highlands of Chincha province, Ica, Peru.

**Materials and Methods:**

A structured questionnaire was used to gather data from 82 goat breeders in three districts: San Juan de Yanac, San Pedro de Huacarpana, and Chavín. Factor analysis of mixed data and hierarchical classification analysis were conducted to identify typologies of goat production systems using R version 4.4.2.

**Results:**

Four distinct goat production types were identified, primarily differentiated by feeding location and deworming frequency. Type 2 (41.5%) was the most prevalent, characterized by grazing on breeders own land, minimal milk production (<1 liter/day, 91.2%), and a focus on cheese and goat kid sales (70.6%). Breeders were predominantly women, with limited resources and extensive management systems. Across all types, mixed breeding was common, and economic reliance on livestock and agriculture prevailed.

**Conclusion:**

Despite their diversity, all goat production systems shared extensive management practices and resource constraints, resulting in low productivity. These findings highlight the need for targeted public policies to improve productivity and sustainability in goat farming within the Ica region.

## INTRODUCTION

Locally available animal genetic resources are the basis for the productivity and adaptability of production systems [1]. Therefore, characterizing these systems and their diversity is the first step in establishing sustainable livestock development policies [2]. Goat production systems worldwide are mainly characterized by being resource-poor, extensive, and mixed [3, 4]. However, since domestication, the unique advantages of goats, particularly Creole goats, have been highlighted compared to other livestock species [5–7]. With their mobile upper lip and greater cellulose digestion efficiency, goats can forage from a wide variety of plants that neither sheep nor cattle can eat [1]. Therefore, they are more adapted to survive in adverse environments [8] and on a small scale [6]. Despite limited resources, goats efficiently convert poor-quality animal feed into good-quality milk and meat [9].

Creole goats play a significant role in the rural economy by providing income, employment, and savings for small-scale breeders [10–14]. In addition to being considered more resilient to climate change [15], it has a high potential to contribute to the achievement of food security and sustainability [16–18]. Despite these competitive advantages, goats often receive less attention in national development programs [1], with policymakers typically focusing on improving cattle, sheep, and South American camelid farming to the detriment of goats [19]. In addition, few studies are available on goat production systems in Peru [20, 21]. In the current context in which poverty reduction and food security are the main challenges for developing countries, implementing public strategies to improve traditional goat farming would significantly benefit rural smallholders [9]. This requires a thorough understanding of the characteristics of local production systems [22]. In this sense, the typological approach, which utilizes multivariate statistical analysis, emerges as a valuable tool for understanding the diversity of livestock production systems and the role of zoogenetic resources within them [1, 23]. Such an approach helps evaluate the structural characteristics of these systems [24] and their ability to cope with changes in the biophysical and socioeconomic conditions in which they operate [25, 26] and to propose alternatives for improvements [9].

Therefore, this study aimed to characterize, through a typology, the diversity of goat production systems in the Highlands of Chincha province, Ica region, Peru.

## MATERIALS AND METHODS

### Ethical approval and Informed consent

This study was based on interviews with goat breeders so ethical approval was not required. Before conducting the surveys, all volunteers signed an informed consent form.

### Study period and location

**Figure 1:**
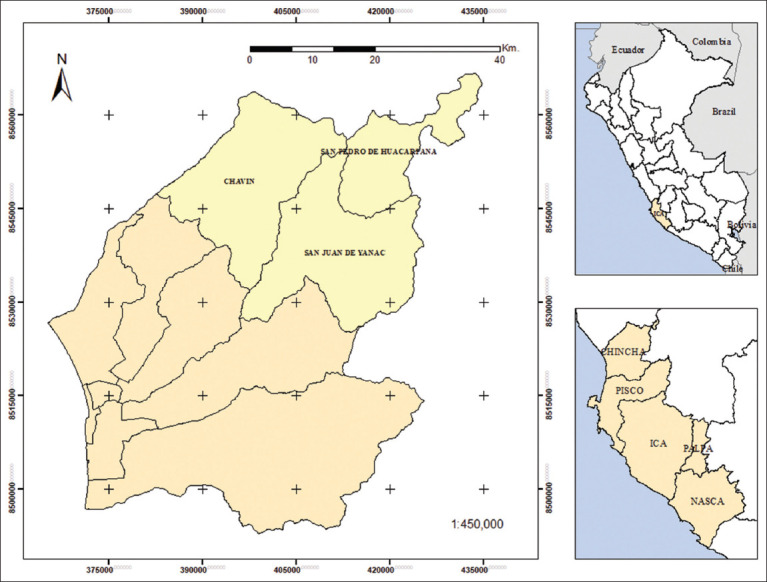
Location of Ica region in Peru country (above right); subdivision of Ica region (below right) and evaluated districts (cream) in Chincha province (left) [Source: The map was generated with ArcGIS 10.8].

The study was conducted from June to August 2023 in the districts of San Juan de Yanac (2533 masl, 13°12′39″S 75°47′13″ W), San Pedro de Huacarpana (3776 masl, 13°02′56″S 75°38′52″ W), and Chavín (3170 masl, 13°04′37″S 75°54′47″ W), located in the mountains of Chincha province, Ica region, Peru (Figure 1). According to the INEI [27], these districts are home to 60% of the goat population in Chincha; predominantly Creole, are raised under an extensive system, and have a diet based mainly on stubble and natural grass.

### Data collection

Structured individual interviews were conducted with 82 goat breeders in different population centers in the selected districts, either in their local dialect or with the support of a translator. For the choice of breeders, the possibilities of geographical access, support from public institutions for their identification and location, predisposition of breeders, and availability of transport were considered. The questionnaire included open and closed questions that considered socioeconomic aspects of the family unit, herd composition, zootechnical management, selection criteria, and commercialization.

### Statistical analysis

**Figure 2:**
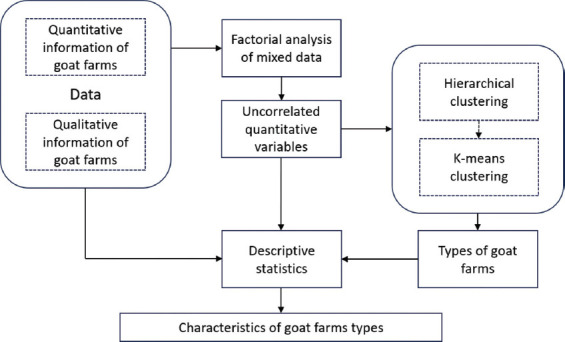
Process of goat farm typification.

All statistical analyses were conducted using R Studio 4.4.2 software to ensure comprehensive evaluation and reproducibility of results. Descriptive statistics, including means, standard deviations, and frequencies, were used to summarize the socioeconomic and management characteristics of goat breeders. To explore the diversity of goat production systems, a factor analysis of mixed data (FAMD) was applied to integrate both qualitative and quantitative variables, reducing dimensionality while preserving variability [28, 29]. The process of typifying goat farms is outlined in Figure 2, which illustrates the analytical flow from data reduction to cluster formation. The principal components derived from FAMD were subjected to hierarchical clustering analysis using Ward’s method to identify distinct typologies of goat herds [30, 31].

Differences among typologies were assessed using a one-way analysis of variance for continuous variables and the Kruskal-Wallis test, where assumptions of normality were violated. Chi-square tests were applied to categorical variables to identify significant associations with typologies.

The variables used in the multivariate statistical analysis are detailed in Table 1, highlighting their importance in characterizing the goat production systems. Statistical significance was determined at p < 0.05, and results are presented using publication-quality tables and visualizations.

**Table 1:** Variables used in the multivariate statistical analysis of the goat production system in the Highlands of Chincha.

No.	Variable	Code	Category and/or value
1	District	DIST	(1) San Juan de Yanac, (2) San Pedro de Huacarpana, (3) Chavín
2	Educational level	GRADO_INSTR	(1) No studies, (2) Prim. Incomplete, (3) Prim. Complete, (4) Sect. Incomplete, (5) Sec. Complete, (6) Sup. Incomplete, (7) Sup. Complete
3	Breeding decisions	DECIS_CRIA	(1) Father, (2) Mother, (3) Both, (4) Other
4	Years of animal husbandry	AÑO_CRIA	(1) <5 years, (2) 5–10 years, (3) 10–20 years, (4) >20 years
5	Main sources of income	FUENT_INGR	(1) Agriculture (2) Livestock, (3) Agriculture and livestock.
6	The main objective of husbandry	OBJE_CRIA	(1) Milk, (2) Dairy Products, (3) Dual Purpose
7	Time dedicated to husbandry	DEDIC_CRIA	(1) 3–6 h, (2) 6–9 h, (3) >9 h
8	Mixed husbandry	CRIA_MIX	(1) Yes, (2) No
9	Goat Feeding	ALIM_CABRA	(1) Grazing, (2) Grazing and Foraging
10	Goat feeding place	LUGAR_ALIM	(1) Own, (2) Communal, (3) Lease, (4) Own and Communal, (5) Own and Lease
11	Number of offspring per delivery	CRIAS_PARTO	(1) Single, (2) Double, (3) Triple
12	Production per goat per day	PROD_CABRA	(1) <1 L, (2) 1–2 L
13	Deworming frequency per year	FREC_DESPARAS	(1) 3 times a year; (2) 2 times a year; (3) 1 time a year; (4) No deworming
14	Sale of milk and/or dairy products	LECHE_DERIV	(1) Cheese, (2) Milk and cheese, (6) Cheese and butter
15	Sale of meat and/or meat derivatives	CARNE_DERIV	(1) Culled goats, (2) Weaned goat kids, (3) Culled goats and weaned goat kids, (4) did not sell
16	Age of the breeder	PROD_AGE	---
17	Percentage of goats that milked	PORCENT_ORDEÑO	---
18	Months of milk production	MESES_PROD	---
19	The goat population in the herd	CABE_CAPRIN	---

## RESULTS

### Socioeconomic characteristics of goat breeders

It was found that 56.1% of the interviewers were men. Similarly, of the total number of respondents, 6.1% were illiterate, 85.4% had completed elementary or secondary school, and only 8.5% had a higher degree. The ages ranged from 24 to 83 years, with a median age of 53. The fathers and mothers of the households made the decisions regarding the upbringing of their goats in 64.6% of agricultural units. 65.9% of breeders have their primary economic income from livestock and agriculture. Of the goat breeders, 73.2% had been breeding for more than 20 years, 65.9% were breeding for milk and meat, and 31.7% were solely focused on producing dairy products, primarily cheeses.

### Management and production characteristics of goatherds

In the Highlands of Chincha, most goat breeders (89.0%) carried out mixed breeding, mainly with sheep, followed by cattle. In the same way, about 90% of the respondents mentioned that they practiced grazing, either with cultivated or natural pastures; the remaining respondents engaged in grazing combined with stubble. The breeders had an average population of 61.4 goats (range = 12 to 367, median = 35), of which 36.1% were goats in production. Their goats yielded milk for 4.1 months on average (median = 4, range = 3 to 6), with 73.2% of the producers reporting that their goats produced less than 1 L of milk per day.

### Typology of goat production systems

**Figure 3:**
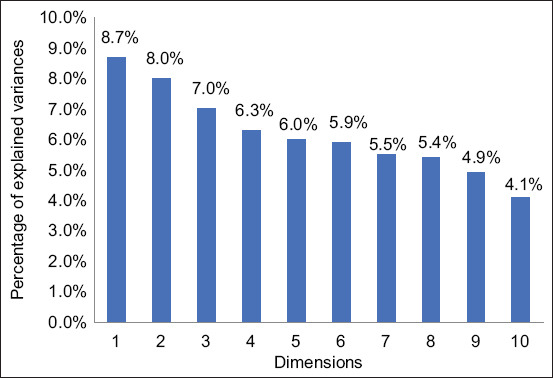
Contribution of the top 10 dimensions to total inertia.

Mixed data factor analysis yielded 29 principal orthogonal components, of which the first 10 explain 62.0% of the total variability observed in the study (Figure 3). However, for the purposes of this study, an explanation will focus on the first two dimensions.

**Figure 4:**
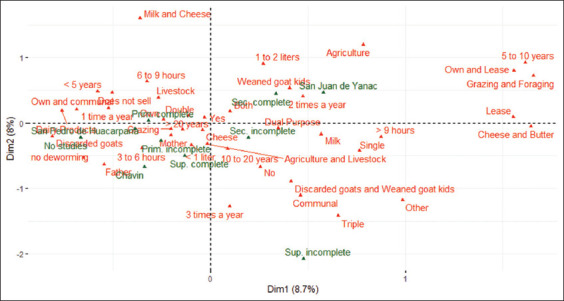
Modalities associated with axes 1 and 2 of the mixed data factor analysis applied to the typology of goatherds in the Highlands of Chincha.

The findings of the factor analysis of the quantitative and qualitative data on goatherds are shown in Figure 4. The first axis (dimension 1) retained 8.7% of the accumulated inertia and showed a high positive correlation with the variable feeding place of the goats. In this sense, it can be mentioned that breeders who have between 5 and 10 years of experience in the breeding of goats have their own feeding area (mainly stubble), and at certain times of the year, they rent to carry out grazing and thus be able to maintain the production of cheeses in their majority, followed by few breeders who also make butter.

On the other hand, dimension 2 (which retains 8% of the total variability) has a high correlation with the variables for the commercialization of meat and/or dairy products and the frequency of deworming. In this regard, it should be noted that breeders who work with goats for 6–9 h a day mostly make their living from livestock. In the same way, breeders who do not perform mixed breeding focus more on the sale of culled goats and weaned goats and, therefore, try to select goats that have more calves at birth.

**Figure 5:**
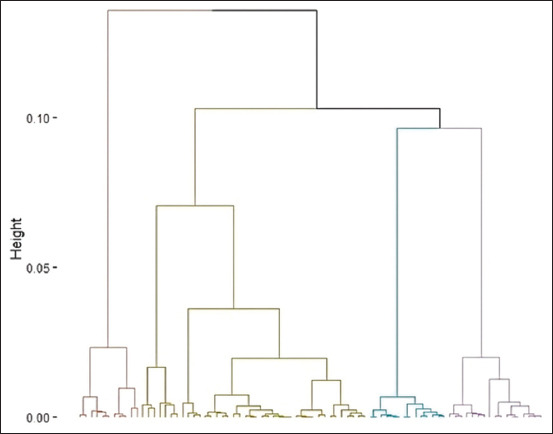
Dendrogram obtained from cluster analysis.

**Figure 6:**
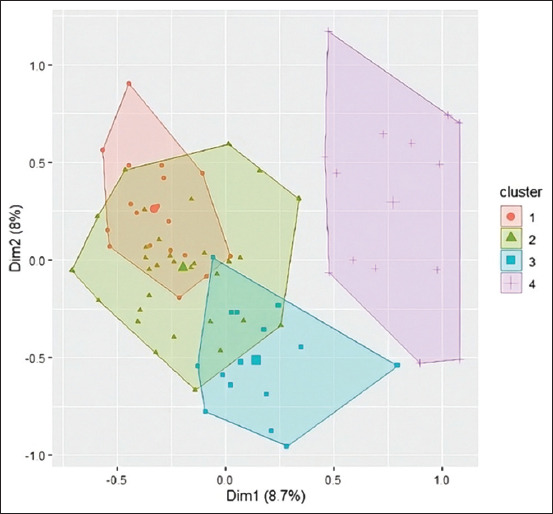
Graphic representation of groups formed in the first two dimensions.

**Figure 7:**
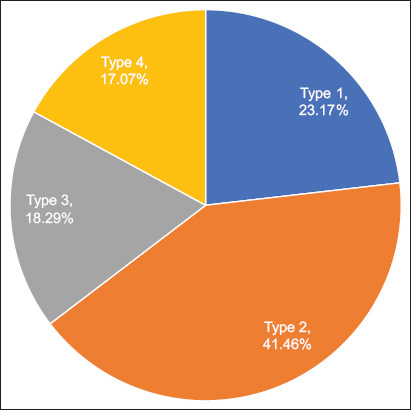
Proportion of different types of goat farms in the Highlands of Chincha.

Subsequently, four distinct goatherd types were identified in the Highlands of Chincha using the breeder’s coordinates on the main axes for cluster analysis (Figures 5 and 6). It is worth noting that the variables “Feeding place” and “Deworming frequency” are the ones that most characterized the partition into the four groups. The proportions of different types of goatherds are shown in Figure 7.

In the first group (n = 19), 57.9% were San Juan de Yanac breeders, followed by 31.6% from San Pedro de Huacarpana. The majority had a full elementary school (36.8%), and both the mother and father of the household made decisions about goat breeding (94.7%). Their main income came from livestock (63.2%), they grazed (100%) on their own and communal land (57.9%), they deworm their goats generally once a year (74.7%), they dedicate 6–9 h to their goats (68.4%), and they produce between 1 and 2 L/day (57.9%) to manufacture, mainly dairy products (73.7%).

In the second group (n = 34), 52.9% were San Pedro de Huacarpana breeders, followed by 32.4% from Chavín. The majority had completed secondary school (32.4%), and the wife was the one who made the decisions about raising goats (50%). Their main economic income came from agriculture and livestock (85.3%); they carried out only grazing (100%); on their land (67.6%), they dewormed their goats generally twice a year (52.9%); and they dedicate 3–6 h to their goats (67.6%) and produce <1 L/day (91.2%), with a tendency to double purpose (70.6%).

In the third group (n = 15), 46.7% were San Pedro de Huacarpana breeders, followed by 33.3% from Chavín. The majority had an incomplete secondary school (46.7%); both the mother and father of the household made decisions about goat breeding (80.0%); their main economic income came from agriculture and livestock (80.0%); they mainly grazed (93.3%); on communal land (53.3%), they deworm their goats generally three times a year (66.7%); they dedicate 3–6 h and more than 9 h to their goats (86.7%); and they produce <1 L/day (100%), with a tendency to double purpose (80%).

In the fourth group (14 breeders), 92.9% of the breeders were from San Juan de Yanac, and the rest were from San Pedro de Huacarpana. The majority had a complete secondary education (57.1%), and both mother and father made decisions about goat breeding (78.6%). Their main economic income came from agriculture and livestock (50.0%); grazing and stubbed (57.1%); and on leased land (50.0%), they deworm their goats generally twice a year (85.7%), dedicate more than 9 h to their goats (71.4%), and produce equitably between <1 L and 1–2 L/day, with the aim of double purpose (92.9%).

Table 2 presents the means ± standard deviations of the quantitative variables considered in the FAMD according to the type of goatherd. In this sense, it can be seen that there were no significant differences between the age of the breeders and the production time of the goats according to the types of herds that were found. On the other hand, type 2 had the fewest goat heads, and type 3 had the lowest percentage of goats being milked.

**Table 2:** Quantitative variables by goatherd type in the Highlands of Chincha.

Variable	Type of goat farm				p-value

	1	2	3	4	
Age of breeder (years)	56.0 ± 15.0^a^	53.8 ± 12.5^a^	56.3 ± 13.2^a^	46.8 ± 10.6^a^	0.1707
Number of goats (unit)	75.5 ± 56.0^ab^	41.5 ± 47.7^b^	53.8 ± 48.3^ab^	98.9 ± 94.3^a^	0.0192
Percentage of milking (%)	33.9 ± 15.5^ab^	36.4 ± 12.5^ab^	30.1 ± 13.6^b^	44.5 ± 15.4^a^	0.0462
Production time (months)	3.9 ± 0.8^a^	4.0 ± 0.8^a^	4.1 ± 0.9^a^	4.5 ± 0.9^a^	0.2575

Different superscripts in each row mean statistically significant differences (p < 0.05).

## DISCUSSION

This study aimed to understand the diversity of goat production systems in the Highlands of Chincha. The findings revealed significant heterogeneity among goatherds in the study region. In this regard, on the central coast of Peru, goat breeding is based on stubble, with reduced herd sizes, low milk production, and low-value meat [20].

A notable observation is the slight majority of male respondents (56.1%) in this study, contrasting sharply with findings from other regions where goat farming characteristics predominantly feature male participants. For instance, in the state of Paraíba in Brazil, 96.8% of the respondents were men [32]; in the Laghouat area of southern Algeria, 99.1% of the respondents were men [1]; and in the Huerter–Norte region of Costa Rica, 83.25% of the respondents were men [33]. This disparity is attributed to traditional gender roles that have historically limited women’s participation in interviews and meetings, a trend that is evolving in Peru. In the same way, the proportion of breeders without formal education in the current study (6.1%) contrasts significantly with rates reported in Algeria, where 44.3% of breeders were illiterate [1]. On the other hand, only 8.5% of the breeders in this study had access to university studies, mainly due to the location of the evaluated districts from the Chincha coast and limited transportation options.

Another common characteristic among breeders is their advanced age, averaging 53.6 years with a median of 53, consistent with typical demographics in goat production systems [34].

Based on the FAMD, the study identified four types of goatherds, three oriented toward dual purposes, highlighting cheese sales, culled goats, and weaned goats. The remaining type (Type 1) primarily focuses on dairy product sales as its main economic activity, prioritizing cultivated pastures, particularly alfalfa, and achieving goat milk production of 1–2 L/day. It should be emphasized that it is difficult to fully capture the diversity of low-resource livestock production systems, which is a limitation of the present typology [30]. However, extensive goat farming remains predominant and economically significant in mountainous areas [35].

Similarly, small breeders’ manual production methods are limited in output but provide significant benefits in terms of lower production costs [36]. The basis of goat feeding in these systems is grasses and stubble, without the inclusion of grain-based feed [37], with the disadvantage of not controlling mating since the animals are released to graze or stubble during the day [38].

In this context, implementing conservation and genetic improvement programs is crucial, yet their sustainability in low-resource production systems with local breeds depends largely on breeder interest shaped by the socioeconomic context [39]. However, based on the participatory interview, it is very likely that breeders will respond favorably [40]. To this end, it should be noted that the use of exotic breeds in this type of production system is not recommended by Husson *et al*. [30], mainly because of their higher nutritional demand, poor adaptability, and low production efficiency [41].

## CONCLUSION

This typological study has delineated four distinct types of goatherds based on their feeding locations and deworming practices. Despite their diversity, these herds share common features, such as reliance on grazing, livestock, and agriculture as primary income sources, coupled with mixed breeding methods. Notably, cheese production and sale of goat kids are the principal economic activities underscored by traditional management practices. The herds are of moderate size, with a significant proportion of goats that milk <1 L of milk daily. These findings provide valuable insights into the heterogeneous nature of goat production systems in the Highlands of Chincha, offering a foundation for targeted interventions to enhance sustainability and productivity.

## AUTHORS’ CONTRIBUTIONS

DJG: Planned and designed the study and revised the manuscript. EAS: Sampling, analyzed the results, and drafted and revised the manuscript. FCT, WPG, JAC: Analyzed the results and revised the manuscript. All authors have read and approved the final manuscript.

## References

[1] Laouadi M, Tennah S, Kafidi N, Antoine-Moussiaux N, Moula N (2018). A basic characterization of small-holders'goat production systems in Laghouat area, Algeria. Pastoralism.

[2] Ruiz F.A, Castel J.M, Mena Y, Camúñez J, González-Redondo P (2008). Application of technico-economic analysis for characterizing, making diagnoses and improving pastoral dairy goat systems in Andalusia (Spain). Small Rumin. Res.

[3] Escareño L, Salinas-Gonzalez H, Wurzinger M, Iñiguez L, Sölkner J, Meza-Herrera C (2013). Dairy goat production systems:Status quo, perspectives and challenges. Trop. Anim. Health Prod.

[4] Madani T, Sahraoui H, Benmakhlouf H (2015). L'élevage caprin en Algérie:Systèmes d'élevage, performances et mutations. In:Workshop national sur “Valorisation des races locales ovines et caprines àfaibles effectifs”, INRA “Institut National de la Recherche Agronomique d'Algérie”, Ministère de l'Agriculture, du Développement Rural et de la Pèche, Alger, Algérie.

[5] Skapetas B, Bampidis V (2016). Goat production in the World:Present situation and trends. Livest. Res. Rural Dev.

[6] Darcan N.K, Silanikove N (2018). The advantages of goats for future adaptation to Climate Change:A conceptual overview. Small Rumin. Res.

[7] Mazhangara I.R, Chivandi E, Mupangwa J.F, Muchenje V (2019). The potential of goat meat in the red meat industry. Sustainability.

[8] Jansen C, van den Burg K (2004). L'élevage Des Chèvres Sous Les Tropiques.

[9] Houessou S.O, Vanvanhossou S.F.U, Yassegoungbe F.P, Adenile A.D, Dahouda M, Guimaraes V.P, Dossa L.H (2021). A typological characterization of rural goat production systems of Benin prior to their sustainability assessment. Arch. Zootec.

[10] Madsen J, Nielsen M.O, Henriksen J (2007). Use of Goats in Poverty Alleviation and Potential Effects on the Environment. Community of Practice (CoP), Copenhagen, Denmark.

[11] Desta H, Alemu B, Kinati W, Mulem A.A, van Eerdewijk A, Wieland B (2020). Contribution of small ruminants to food security for Ethiopian smallholder farmers. Small Rumin. Res.

[12] Monau P, Raphaka K, Zvinorova-Chimboza P, Gondwe T (2020). Sustainable utilization of indigenous goats in Southern Africa. Diversity.

[13] Sow F, Camara Y, Traore E.H, Cabaraux J.F, Missohou A, Antoine-Moussiaux N, Hornick J.L, Moula N (2021). Characterisation of smallholders'goat production systems in the Fatick area, Senegal. Pastoralism.

[14] Behingan M.B, Houndonougbo P.V, Alowanou G.G, Koudande D.O, Chrysostome A.A.M C (2023). Characterization and typology of goat farm production systems in Benin. Mor. J. Agric. Sci.

[15] Pragna P, Surinder S.C, Veerasamy S, Leury B.J, Dunshea F.R (2018). Climate change and goat production:Enteric methane emission and its mitigation. Animals (Basel).

[16] Capote J (2017). Introductory Chapter:Goats in arid and mountain areas. In:Sustainable Goat Production in Adverse Environments.

[17] Kwashirai V, Mhike I (2019). Green or grey?Goats, economy and ecology in Nkayi district, Zimbabwe:1980–2017. Glob. Environ.

[18] Akounda B, Ouédraogo D, Burger P.A, Rosen B.D, Van Tassell C.P, Sölkner J, Soudré A (2023). Characterization of goat production systems in two agro-ecological zones of Burkina Faso, West Africa. Int. J. Environ. Agric. Biotechnol.

[19] Mueller J.P, Rischkowsky B, Haile A, Philipsson J, Mwai O, Besbes B, Valle Zárate A, Tibbo M, Mirkena T, Duguma G, Sölkner J, Wurzinger M (2015). Community based livestock breeding programs:Essentials and examples. J. Anim. Breed. Genet.

[20] Sarria J.A, Navia G (2014). Description of the goat production system and guidelines for a development proposal in the Cañete valley [Caracterización del sistema de producción caprina y lineamientos de una propuesta de desarrollo en el valle de Cañete]. An. Cient.

[21] Temoche V.A (2019). Sistema de Producción De Caprinos en Tres Zonas Vulnerables al Cambio Climático de la Región Piura. Master's Thesis.

[22] FAO (2012). Phenotypic Characterization of Animal Genetic Resources.

[23] Alvarez S, Timler C.J, Michalscheck M, Paas W, Descheemaeker K, Tittonell P, Andersson J.A, Groot J.C (2018). Capturing farm diversity with hypothesis-based typologies:An innovative methodological framework for farming system typology development. PLoS One.

[24] Blanco-Penedo I, Sjöström K, Jones P, Krieger M, Duval J, van Soest F, Sundrum A, Emanuelson U (2019). Structural characteristics of organic dairy farms in four European countries and their association with the implementation of animal health plans. Agric. Syst.

[25] Friedman R, Hirons M.A, Boyd E (2019). Vulnerability of Ghanaian women cocoa farmers to climate change:A typology. Clim. Dev.

[26] Tittonell P, Bruzzone O, Solano-Hernández A, López-Ridaura S, Easdale M.H (2020). Functional farm household typologies through archetypal responses to disturbances. Agric. Syst.

[27] INEI (2012). Base de Datos del IV CENAGRO.

[28] Lê S, Josse J, Husson F (2008). FactoMineR:An R package for multivariate analysis. J. Stat. Softw.

[29] Kassambara A, Mundt F (2020). Factoextra:Extract and Visualize the Results of Multivariate Data Analyses. R Package Version 1.0.7.

[30] Husson F, Josse J, Pages J (2010). Principal Component Methods-Hierarchical Clustering-Partitional Clustering:Why Would we Need to Choose for Visualizing Data?. Agrocampus Ouest, Applied Mathematics Department, Rennes.

[31] Mugumaarhahama Y, Ayagirwe R.B.B, Mutwedu V.B, Cirezi N.C, Wasso D.S, Azine P.C, Karume K (2021). Characterization of smallholder cattle production systems in South-Kivu province, eastern Democratic Republic of Congo. Pastoralism.

[32] de Figueiredo R, Lima A.M, Alves J.R, da Costa D.F, Pinheiro R.R, Fernandes F.S, Santos S, Alves C.J (2017). Characterization and typology of sheep and goat production systems in the State of Paraíba, a semi-arid region of northeastern Brazil. Semin. Cienc. Agrar.

[33] Barboza M.A, Jiménez J.P, Porras Á.J, Miranda O, Camacho M.I (2020). Characterization of Goat Production Systems in the North Huetar Region of Costa Rica using Multivariate Statistical Techniques. Universidad Nacional, Costa Rica.

[34] Gökdai A, Magrin L, Sakarya E, Contiero B, Gottardo F (2020). Characterization and typologies of dairy goat farms in the Mediterranean region:A case of Italy and Turkey. Small Rumin. Res.

[35] Ruiz F.A, Vázquez M, Camuñez J.A, Castel J.M, Mena Y (2020). Characterization and challenges of livestock farming in Mediterranean protected mountain areas (Sierra Nevada, Spain). Span. J. Agric. Res.

[36] Hemme T, Otte J (2010). Status of and Prospects for Smallholder Milk Production:A Global Perspective. Food and Agriculture Organization of the United Nations, Rome.

[37] Maleko D, Msalya G, Mwilawa A, Pasape L, Mtei K (2018). Smallholder dairy cattle feeding technologies and practices in Tanzania:Failures, successes, challenges and prospects for sustainability. Int. J. Agric. Sustain.

[38] Tindano K, Moula N, Traoré A, Leroy P, Antoine-Moussiaux N (2017). Assessing the diversity of preferences of suburban smallholder sheep keepers for breeding rams in Ouagadougou, Burkina Faso. Trop. Anim. Health Prod.

[39] Biscarini F, Nicolazzi E, Alessandra S, Boettcher P, Gandini G (2015). Challenges and opportunities in genetic improvement of local livestock breeds. Front. Genet.

[40] Manirakiza J, Hatungumukama G, Besbes B, Detilleux J (2020). Characteristics of smallholders'goat production systems and effect of Boer crossbreeding on body measurements of goats in Burundi. Pastoralism.

[41] Kahi A. K, Thorpe W, Nitter G, Van Arendonk J.A.M, Gall C.F (2000). Economic evaluation of crossbreeding for dairy production in a pasture-based production system in Kenya. Livest. Prod. Sci.

